# Robot-Assisted Laparoscopic Resection With the Transanal Approach for Massive Rectal Gastrointestinal Stromal Tumor: A Case Report

**DOI:** 10.7759/cureus.76352

**Published:** 2024-12-24

**Authors:** Yusuke Yoshida, Fuminori Teraishi, Ryohei Shoji, Yuki Matsumi, Toshiyoshi Fujiwara

**Affiliations:** 1 Department of Gastroenterological Surgery, Okayama University Graduate School of Medicine, Dentistry, and Pharmaceutical Sciences, Okayama, JPN; 2 Department of Gastroenterological Surgery, Okayama University Hospital, Okayama, JPN

**Keywords:** bi-directional approach, locally advanced tumor, rectal gist, robot-assisted surgery, tatme

## Abstract

Rectal gastrointestinal stromal tumors (GISTs) are often asymptomatic and may be detected as giant tumors. This may require highly invasive surgery for radical resection. Here, we describe a 74-year-old man with a locally advanced non-metastatic GIST in the right anterolateral wall of the lower rectum. The tumor was giant (128 × 93 mm), and invasion into adjacent organs (right seminal vesicle and prostate gland) was suspected. Although neoadjuvant chemotherapy (NAC) with imatinib reduced the tumor size, it was still giant, 80 mm in diameter. Therefore, we performed super-low anterior resection using a robot-assisted laparoscopic approach with the transanal approach. The bi-directional approach enabled safe and precise surgery, is expected to increase the rate of anorectal preservation as well as R0 resection, and may prevent a decline in quality of life.

## Introduction

Gastrointestinal stromal tumors (GISTs) are mesenchymal tumors that occur throughout the gastrointestinal tract, but rectal GISTs are relatively rare, accounting for 3-5% of those [1,2]. Rectal GISTs are rarely symptomatic and are not uncommonly discovered as giant tumors [3]. The giant pelvic tumors can impair pelvic maneuverability and may require extensive surgery to cure the tumors. Here, we report a case of giant GIST of the right anterolateral wall of the lower rectum removed by robot-assisted laparoscopic ultra-low anterior resection with a transanal approach.

## Case presentation

**Figure 1:**
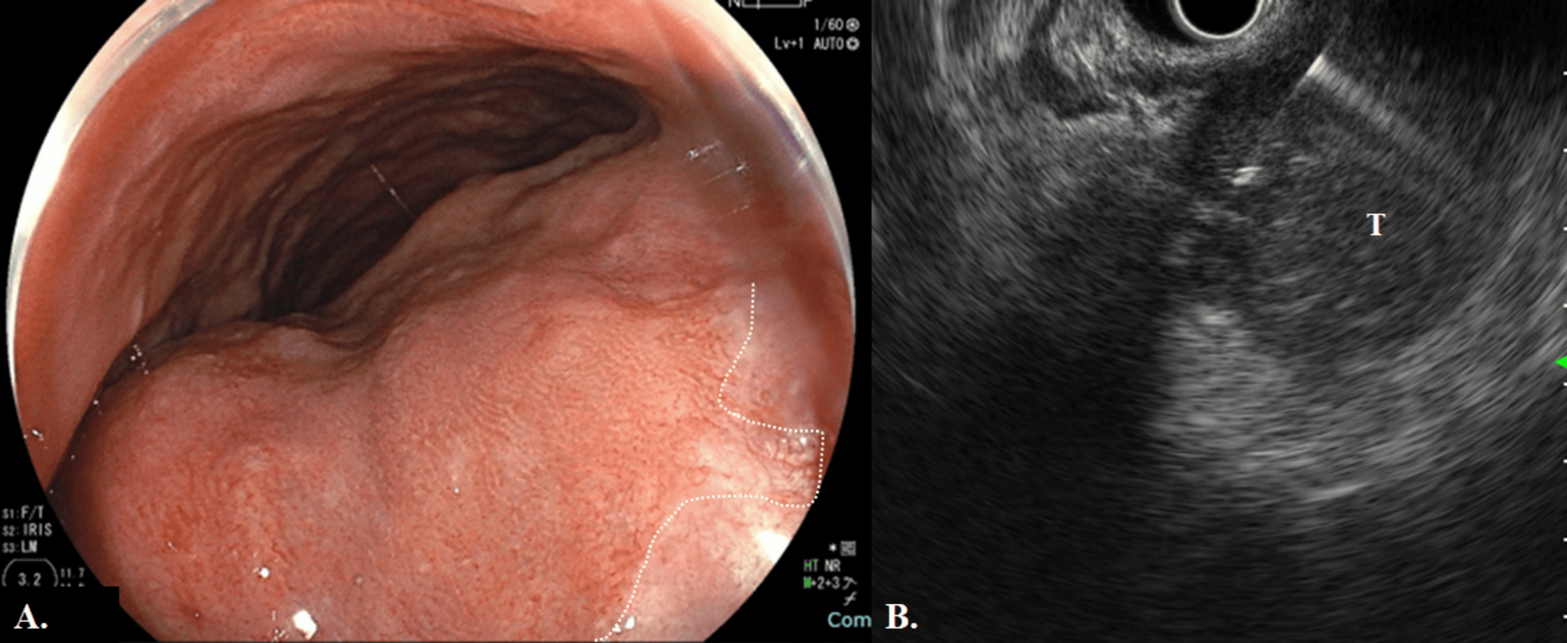
Colonoscopy and endoscopic ultrasound images (A) Pretreatment colonoscopy showed extramural compression of the lower rectum (Rb). The distance of the lesion from the anal verge was about 3 cm, and the lower margin of the lesion was located as high as the dentate line (white dotted line). (B) Endoscopic ultrasound-guided fine needle aspiration was performed at another hospital. T, tumor

A 74-year-old man visited the urology department of another hospital with a chief complaint of frequent urination, and magnetic resonance imaging (MRI) indicated a pelvic tumor. A colonoscopy showed extramural extension of the rectum, and endoscopic ultrasound-guided fine needle aspiration revealed a diagnosis of GIST (Figure 1).

**Figure 2:**
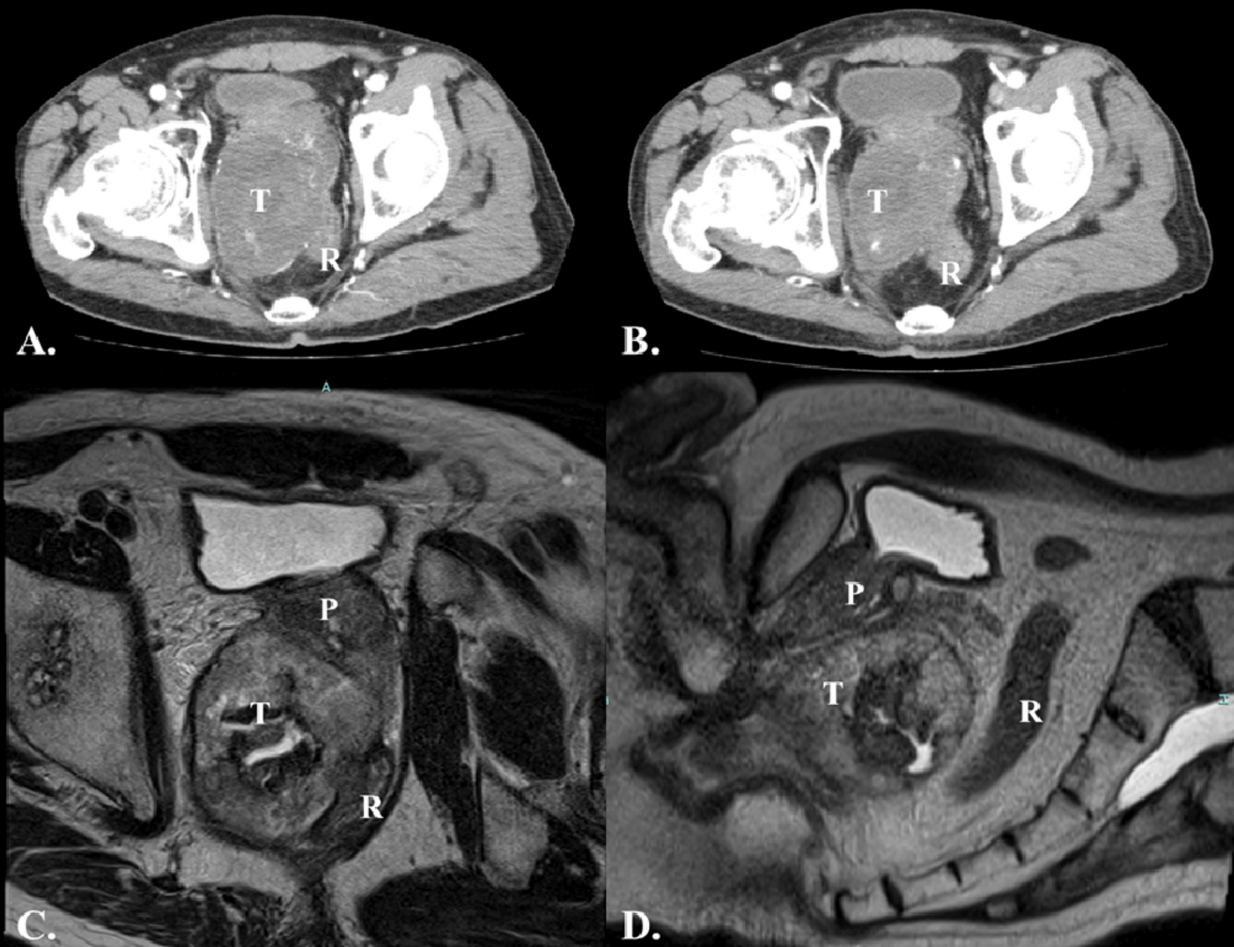
Pretreatment computed tomography (CT) images and post-neoadjuvant imatinib treatment images (A) Pretreatment contrast-enhanced CT shows the tumor (128 mm in diameter) is present in the right anterolateral wall of the rectum. (B) Contrast-enhanced CT shows tumor shrinkage (80 mm in diameter). (C,D) Magnetic resonance imaging shows rectal compression by the tumor and contact with the prostate, with a slight border between the tumor and the prostate. (B-D) Post-neoadjuvant imatinib treatment images. P, prostate; R, rectum; T, tumor

Neoadjuvant chemotherapy (NAC) (imatinib mesylate 400 mg/day) was initiated because the tumor was large (128 × 93 mm), and invasion into adjacent organs was suspected. After two years of treatment, the tumor had shrunk to 80 mm, but edema and muscle pain in the lower limbs worsened, leading to the decision to operate and referral to our hospital. Preoperative imaging assessment predicted that the patient would have difficulty with prostate dissection by a laparoscopic approach alone, so we decided to combine a robot-assisted approach with articulated features and a transanal approach (transanal total mesorectal excision [taTME]) to ensure negative margins (Figure 2).

**Figure 3:**
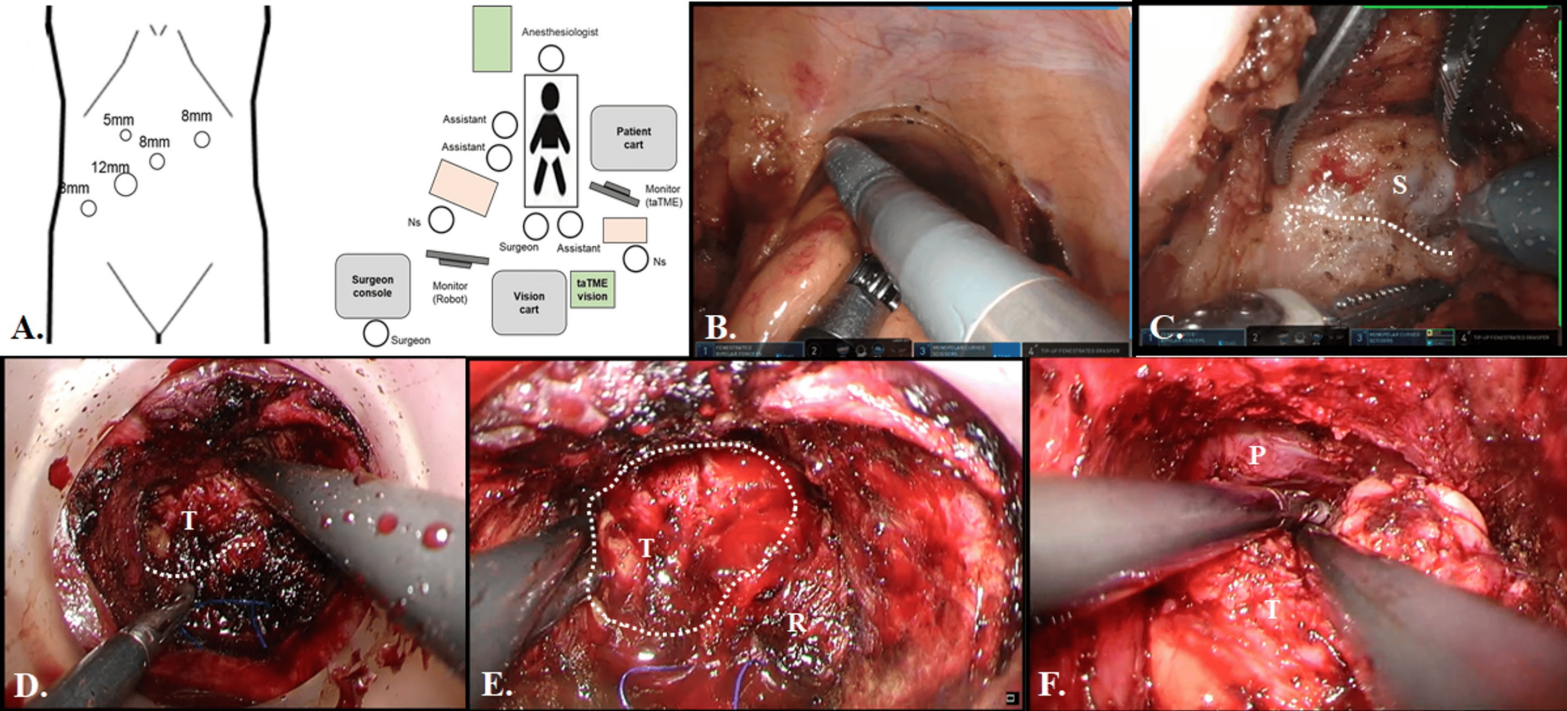
Schematic of port and equipment placement and Intraoperative images of rectal dissection (A) Original illustration by the author. (B,C) Robot-assisted laparoscopic views. The peritoneum was incised cephalad of the peritoneal inversion, and dissection was performed to the level of the seminal vesicle. (D) The caudal side of the tumor was identified. (E) The rectum was compressed posteriorly to the left by the tumor. (F) Dissection was performed to the level of the prostate. (D-F) Transanal views. S, seminal vesicle T, tumor R, rectum P, prostate

The operation was initiated with robot-assisted laparoscopy using five ports (Figure 3). The sigmoid colon was mobilized with a medial-to-lateral approach to preserve the left ureter and gonadal vessels. The inferior mesenteric artery was ligated near its root, and the inferior mesenteric vein and ascending branch of the left colonic artery were ligated at the same level. After dissection of the dorsal rectum, dissection of the ventral rectum was performed. However, due to the displacement of the seminal vesicles and nerves caused by the massive tumor, it was difficult to identify the dissected layer. So, taTME was initiated at this point, and the surgery proceeded in parallel from both directions. The giant tumor (long diameter 80mm) was located in the right anterolateral wall of the rectum. From the robot-assisted laparoscopic side, the seminal vesicle was partially exposed with Denonvilliers’ fascia on the resection side, and dissection was performed on the ventral side of the tumor. From the taTME side, the rectum was deviated to the left side due to tumor compression, but the caudal side of the tumor was recognizable; hence, dissection proceeded so that the tumor was attached to the rectal side. The dorsal side of the rectum was dissected to the level of the endopelvic fascia, and then the right and left lateral ligaments were dissected to reach the pelvic floor. At this point, dissection from the taTME side had progressed to the head of the tumor (Figure 3), and dissection was completed by connecting the dissected layers.

**Figure 4:**
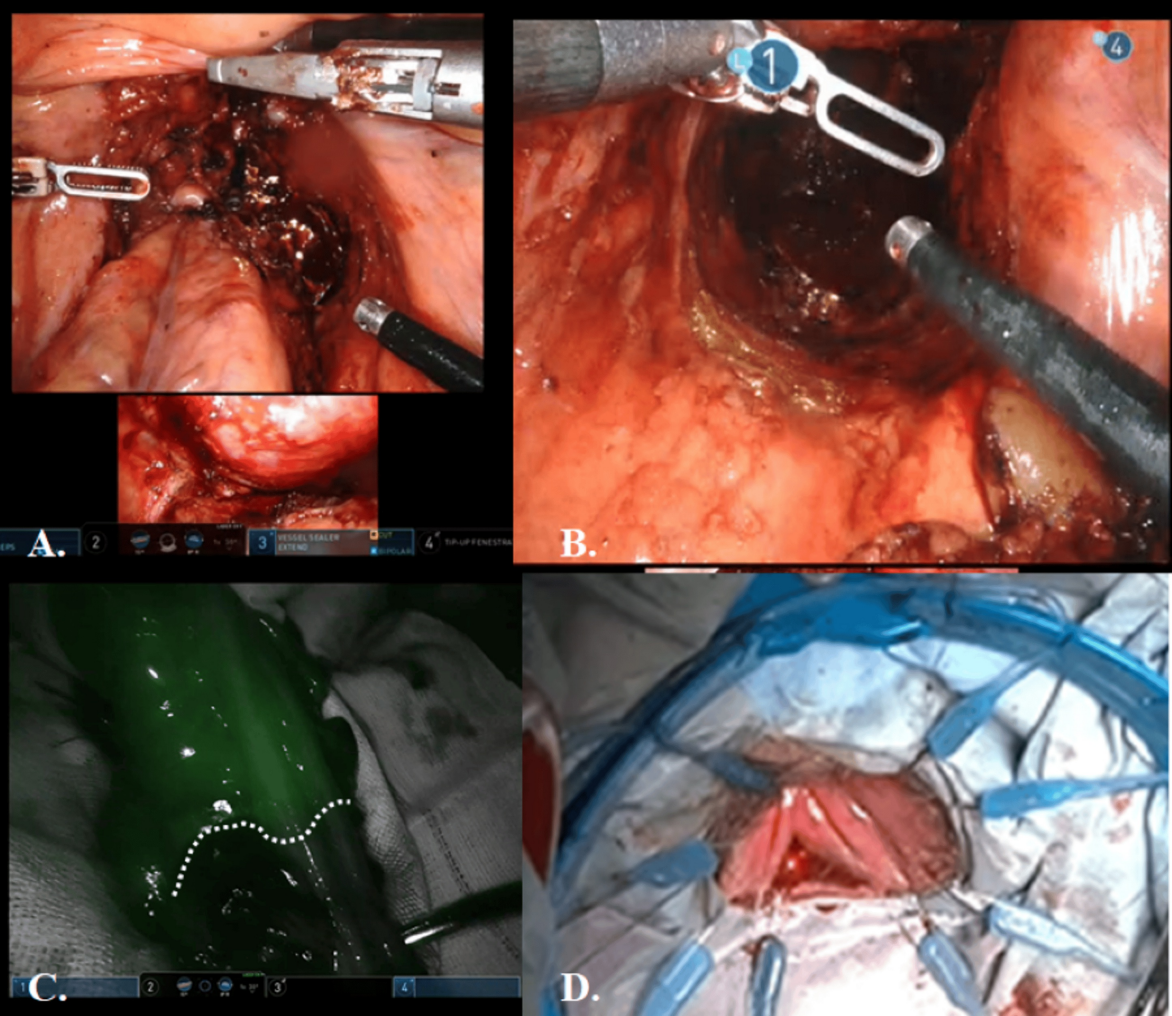
Intraoperative images of the opening of the dissection line from both directions and reconstruction. (A) Dissection was also completed from the abdominal side to the level of the prostate. The tumor and prostate were dissected from the transanal side, and the dissected layers were continued from the patient’s right side on the abdominal and transanal sides. (B) The dissection was completed with a full circumferential spread. (C) After elevation of the intestine outside the body, blood flow in the remaining intestine was checked with ICG, and the specimen was removed. (D) Reconstruction was performed by sigmoid colon-anal anastomosis (hand sewing).

The sigmoid colon to the rectum was elevated outside the body. After confirming intestinal blood flow using indocyanine green (ICG), the intestinal tract was dissected, and the specimen was removed. A hand-sewn sigmoid colon-anal anastomosis was performed (Figure 4).

**Figure 5:**
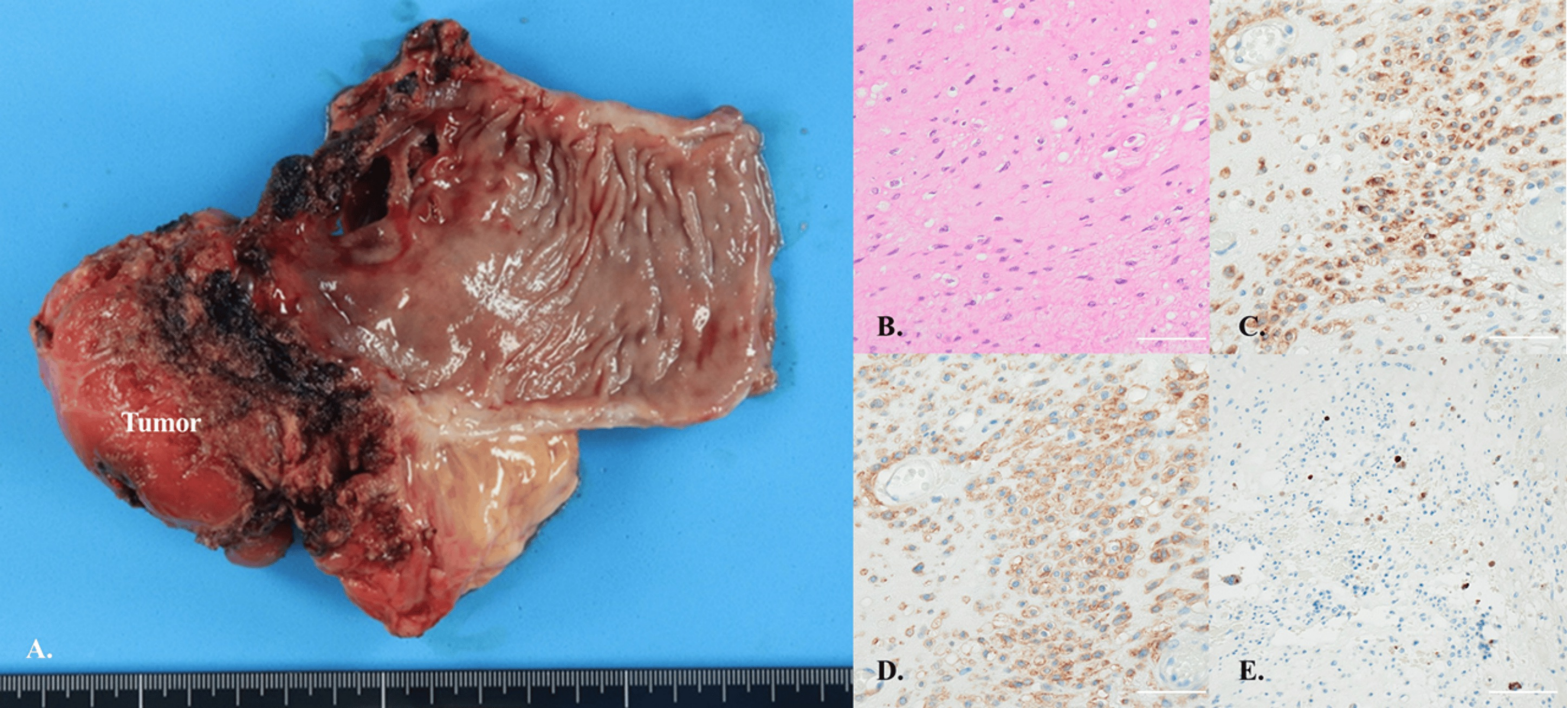
Macroscopic and histological findings of the specimen (A) The specimen measured 80 × 55 × 50 mm with well-defined borders. (B) Hematoxylin and eosin (H&E) staining. (C-E) Immunohistochemical staining of c-kit, DOG1, and Ki-67, respectively. Scale bars: B-D, 50 μm; E, 100 μm

The operation was completed with a temporary ileostomy. The excised specimen showed complete TME, the distal margin was secured, and the circumferential margin showed no exposed tumor (Figure 4). The patient had a postoperative complication of outlet obstruction, which resolved with conservative treatment, and the patient was discharged on the 16th postoperative day. Stoma closure was performed six months postoperatively after confirming the absence of both anastomotic leakage and local recurrence. Pathology showed a well-defined rectal submucosal tumor with low nuclear atypia and necrosis in some areas. Immunohistochemical staining was positive for c-kit and DOG1, and the diagnosis was GIST with a Ki-67 index of 5-10% (Figure 5).

## Discussion

Surgical resection is the standard treatment for GISTs, although lymph node dissection is not recommended, as lymph node metastases are rare [4]. Pelvic organs, including the rectum, are anatomically restricted, and surgical manipulation is heavily influenced by tumor size and location. In the case of a giant rectal GIST, such as the present case, highly invasive procedures (e.g., abdominoperineal resection or total pelvic resection) may be necessary to ensure adequate dissection [5]. In our case, a giant rectal GIST compressing surrounding organs, such as the prostate, was initially treated with NAC using imatinib to achieve a safe R0 resection. After a certain degree of tumor reduction was achieved, radical resection was performed. The combination of the robot-assisted laparoscopic and transanal approach allowed for safe and precise dissection along the border with the prostate, which was the most challenging part of the resection. Anal preservation was possible simultaneously with R0 resection. There have been reports on the usefulness of transabdominal and transanal bidirectional approaches in the surgery of giant rectal GISTs [6,7], particularly when the tumor is located ventral to the rectum, as in this case.

While radical resection is the goal for rectal GISTs, preservation of the patient’s vital function should also be considered. Jakob et al. [8] reported a high rate of positive margins in 10 out of 21 patients who underwent function-preserving local resection for rectal GISTs with tumor diameters between 10 and 80 mm (median: 40 mm). Depending on tumor size and localization, local resection should be carefully considered. Regarding the approach to rectal GISTs, we believe that a laparoscopic approach is preferable to an open approach due to the limited space and visibility within the pelvic cavity. In cases where the tumor is not located low and is small in size, laparoscopic surgery may be feasible. However, if the tumor is located low, a robotic approach or taTME may be more effective. As in this case, where the tumor is large and located low, we believe that a combination of robotic surgery and taTME is more effective.

Preoperative imatinib therapy for rectal GISTs has been reported to improve R0 resection rate, anal function preservation rate, and prognosis [9,10], making it a promising treatment option for patients who require highly invasive surgery or whose postoperative quality of life may be impaired by extensive resection. However, the efficacy of preoperative therapy has not been fully established. In this case, after two years of treatment with imatinib, some tumor shrinkage was achieved, but the side effects of edema and myalgia in the lower limbs worsened. Therefore, continuing treatment was deemed unprofitable, and surgery was decided upon.

At the time of surgery, it was essential for both the surgeon and the assistant to be aware that the anatomical positions of the prostate and rectum had been deviated due to compression by the giant GIST, as indicated by preoperative imaging findings.

## Conclusions

Even in the case of a giant tumor occupying the pelvic cavity, the present approach may help avoid excessive surgical invasiveness if a radical cure can be achieved through local resection, as seen with GISTs. However, it is important to note that the anatomical positions of the pelvic organs may be displaced due to tumor compression. Additionally, the surgeon must be proficient in evaluating preoperative imaging to determine whether radical resection can be safely performed without compromising neighboring organs. In this case, robot-assisted laparoscopic surgery combined with the transanal approach not only enabled accurate and safe surgery but also preserved the patient’s quality of life.
